# Evaluation of Microvascular Flow Imaging and Power Doppler Ultrasonography in an Equine Model of Induced Superficial Digital Flexor Tendonitis

**DOI:** 10.1111/vru.70226

**Published:** 2026-07-29

**Authors:** Miriam R. Garcia‐Collao, Kyla Ortved, Darko Stefanovski, Joie L. Christensen, Claire Underwood

**Affiliations:** ^1^ Department of Clinical Studies New Bolton Center School of Veterinary Medicine University of Pennsylvania Kennett Square Pennsylvania USA

**Keywords:** healing, horses, tendon, ultrasound, vascularity

## Abstract

Superficial digital flexor tendon (SDFT) injuries are common in athletic horses and are associated with prolonged healing and high reinjury rates. Conventional ultrasonography provides structural information but limited physiologic assessment. This study compared microvascular imaging (MVI) and Power Doppler (PD) for evaluating vascular changes in a collagenase‐induced equine SDFT injury model and assessed the effect of scanning technique on vascular signal detection. Eight adult Thoroughbred horses underwent bilateral collagenase‐induced SDFT injury, with one forelimb randomly assigned to intralesional autologous protein solution treatment and the contralateral limb receiving intralesional saline. Ultrasonographic examinations were performed at baseline and Weeks 0, 2, 4, 8, and 12 post‐injury. Longitudinal and transverse MVI and PD images were evaluated using semiquantitative vascularity scores and quantitative vascularity ratios. Mixed‐effects Poisson regression accounted for repeated measures and assessed effects of imaging modality, time, scanning plane, weight‐bearing (WB) stance, stand‐off use, and observer experience. MVI detected higher vascularity than PD at all post‐injury time points (2.04 [95% confidence intervals—CI: 1.73–2.34] vs. 0.87 [0.64–1.10]) with peak vascularity at Week 2 followed by gradual decline during healing. Quantitative vascularity ratios mirrored semiquantitative findings. Longitudinal imaging yielded greater vascular detection than transverse imaging for both techniques (1.97 [95% CI: 1.72–2.22] vs. 0.92 [0.69–1.15]), whereas WB status and stand‐off pad use had no statistical significant effect. Observer experience influenced vascularity scores but not temporal trends. These findings demonstrate MVI detects more intratendinous vascularity than PD during SDFT injury and healing, and support its use as a complementary tool for monitoring equine tendinopathy.

**Abbreviations**
CSA: cross‐sectional area
DACB: distal to the accessory carpal bone
NSD: non‐stand‐off
NWB: non‐weight‐bearing
SD: stand‐off
WB: weight‐bearing

## 1 Introduction

Superficial digital flexor tendon (SDFT) injuries are among the most prevalent musculoskeletal disorders in athletic horses [1]. The consequences of this pathology are lameness, prolonged rehabilitation times, and a high rate of reinjury that varies from 40%–70% depending on the horse's activity [2, 3, 4]. Tendon healing is often prolonged, and the repaired tissue frequently exhibits inferior biomechanical properties and disorganized structure, which likely contributes to the high rates of reinjury [3, 5]. An accurate diagnosis, proper management, and close monitoring of tendon healing are critical for tailoring rehabilitation protocols and minimizing the risk of recurrence [6].

Accurate assessment of tendon and ligament health is also crucial for effective diagnosis and management. B‐mode ultrasound is widely recognized as the first‐line imaging technique for evaluating the SDFT in horses, as it is a cost‐effective, readily accessible modality, and allows dynamic assessment [7]. It enables real‐time assessment of structural changes that alter echogenicity, fiber alignment, and cross‐sectional area [8]. However, it does not provide physiologic information on prodromal changes within soft tissues, active damage, or the structural integrity of the healing tissue. Therefore, it can be challenging to interpret whether structural changes are active and clinically significant, chronic and inactive, or represent adaptive remodeling [8, 9, 10].

Neovascularization in tendons is associated with active pathology, nerve growth, and pain and represents an important component during the healing process [9, 10, 11]. However, the clinical significance of persistent mild vascularity within chronic lesions is not known. Conventional Doppler imaging techniques, including Color Doppler (CD) and Power Doppler (PD), have been used to assess neovascularization in human and equine tendinopathies [12, 13, 14, 15]. These have limited sensitivity for detecting low‐velocity microvascular flow and are susceptible to motion artifacts [16, 17, 18].

Microvascular imaging (MVI) techniques have been developed to overcome the previously mentioned challenges. These methods use advanced Doppler‐based technologies that employ sophisticated clutter suppression algorithms to separate low‐velocity blood signals from tissue motion artifacts and background noise, enabling visualization of microvessels that are not visible with conventional Doppler imaging [19]. Many vendor‐specific implementations exist, including superb microvascular imaging (SMI; Canon/Toshiba, used in this study), XFlow and MicroV (Esaote), and MicroFlow Imaging (Samsung). Their performance may vary due to differences in underlying processing algorithms and hardware. These techniques can operate in either monochrome or color mode, providing an overlay image that isolates vascular flow from surrounding tissue signals [17]. Semiquantitative and quantitative grading scores have been reported [18]. The application of these techniques provided new opportunity for diagnosing musculoskeletal and rheumatic disorders, monitoring therapeutic outcomes, and improving prognostic assessments [20]. Although MVI has demonstrated superior sensitivity compared with conventional Doppler techniques in human musculoskeletal imaging, direct comparisons between MVI and PD for assessing vascularization during tendon healing remain limited in the human literature [17, 18, 21]. In horses, there are currently no manuscripts describing the use of MVI for the evaluation and monitoring of tendon injuries.

This study aimed to compare MVI and PD in a collagenase‐induced SDFT injury model and evaluate different techniques for image acquisition. The primary hypothesis was that MVI would detect more vascular changes during tendon injury and healing compared to PD. The secondary objective was to evaluate whether different ultrasound imaging plane (longitudinal versus transverse), the presence or absence of a stand‐off pad, and weight‐bearing (WB) versus non‐weight‐bearing (NWB) stance affected MVI and PD signal.

## 2 Methods

### 2.1 Selection and Description of Subjects

#### 2.1.1 Animals

Eight systemically healthy adult Thoroughbred horses, aged 3–8 years (median age = 5 years), including four mares and four geldings, with no history of superficial digital flexor tendinopathy, were enrolled in this study. This horse population has been described in a previous publication [22]. Throughout the study period, horses were maintained on strict stall rest for 8 weeks, followed by 5 min of daily hand‐walking until Week 12. All animal procedures were conducted in accordance with protocols approved by the Institutional Laboratory Animal Care and Use Committee at The University of Pennsylvania.

#### 2.1.2 Tendonitis Induction

Bilateral tendonitis was induced in the forelimbs as previously described [22] using a collagenase injection after ultrasound examination verified no preexisting SDFT abnormalities were present. The collagenase injection protocol was based on Watts et al. [23] with minor modifications [22]. Briefly, horses were sedated intravenously with xylazine (0.4 mg/kg), detomidine (0.01 mg/kg), and acepromazine (0.04 mg/kg). The palmar metacarpus was clipped and aseptically prepared, and local anesthesia was achieved via subcutaneous line block with 2% mepivacaine. Using ultrasound guidance, a 17‐gauge Tuohy epidural needle was introduced through the palmar surface of the limb into the center of the SDFT at a point 12 cm distal to the accessory carpal bone (DACB). The needle was then advanced within the tendon, in a proximal‐to‐distal direction along its long axis, until the tip was visualized ultrasonographically at 18 cm DACB. At this point, collagenase type I (1000 U diluted in 270 µL sterile saline) was injected while withdrawing the needle. The procedure was repeated on the contralateral forelimb, followed by limb bandaging. Phenylbutazone (4.4 mg/kg IV) was administered post‐procedure. Horses were confined to stall rest, received oral anti‐inflammatory therapy, and underwent daily clinical and weekly lameness evaluations during the study [22].

#### 2.1.3 Intralesional Therapy

Intralesional injection of autologous protein solution (APS; Pro‐Stride; Zoetis) was performed in one randomly assigned forelimb of each horse 7 days after induction of tendonitis (defined as Week 0), with the contralateral limb receiving an intralesional saline injection as a control, as previously described [22]. Briefly, local anesthesia was performed using 2% mepivacaine via a subcutaneous line block at the palmar proximal metacarpus. Following aseptic preparation, 3 mL of APS or saline were injected into the lesion at 16 cm DACB under ultrasound guidance using a 22‐gauge, 1.5‐in. needle. Post‐injection care included 7 days of limb bandaging, 8 weeks of strict stall rest, and a gradual return to exercise through 5 min of daily hand‐walking until study termination 12 weeks following treatment.

### 2.2 Data Recording and Analysis

#### 2.2.1 Ultrasonographic Examination

Both forelimb SDFTs were evaluated ultrasonographically by a single experienced sonographer (C.U.) immediately prior to tendinitis induction (Week −1) and at Weeks 0 (treatment), 2, 4, 8, and 12 following treatment. All scans were performed with the horse standing using an Aplio i700 system (Canon, CA) with a 5–18 MHz linear probe.

B‐mode images (longitudinal and transverse) were acquired in the WB stance with a stand‐off (SD) and included a cineloop along the entire length of the tendon and still images acquired from 8 to 30 cm DACB at 2 cm intervals. Nine second cineloops using PD and MVI were obtained at 12, 16, and 20 cm DACB in both longitudinal and transverse planes in WB stance. This area corresponded to the site of collagenase injection and the zone of maximal injury. For technique comparison, PD and MVI cineloops were also acquired without a stand‐off (NSD) and in an NWB stance at 16 cm DACB. Thus, this enables comparison of SD + WB, NSD  + WB, and NSD + NWB at this level. The SD + NWB combination was not performed in this study.

B‐mode image settings were standardized across all examinations at a frame rate of 29 frames/s, with a gain of 83, a frequency of 17 MHz, and a dynamic range of 70. Doppler ultrasound settings were optimized prior to the study to maximize detection of vascular signals within the tendons. PD was performed with a frame rate of 6–14 frames/s, a color gain of 38, a dynamic range of 65–70, a pulse repetition frequency of 1.6 kHz, and a focal depth of 3.25 cm. MVI was acquired using Canon's SMI preset on the Aplio i700 platform, with a frame rate of 16–17 frames/s, with a color gain of 45, a dynamic range of 65–70 dB, a pulse repetition frequency of 1.6 kHz, and a focal depth of 3.25 cm.

#### 2.2.2 Image Analysis

B‐mode images were evaluated for tendon echogenicity (0 = mostly normal; 1 = mostly echogenic; 2 = 50% echogenic and 50% anechoic; 3 = mostly anechoic) and linear fiber pattern (0 = 75%–100% parallel fibers; 1 = 50%–74% parallel fibers; 2 = 25%–49% parallel fibers; 3 = 0%–24% parallel fibers) as previously reported [22].

For analysis of the Doppler signal, frames displaying the maximum amount of flow were extracted from each cineloop and stored as still images (in TIFF format) by the inexperienced observer (M.G.C.). The proportion of tendon exhibiting vascular signals was assessed individually, and only stable vessels were included in the analysis, given that both MVI and PD are susceptible to motion artifacts. The same images were used for both semiquantitative and quantitative analyses.

**FIGURE 1:**
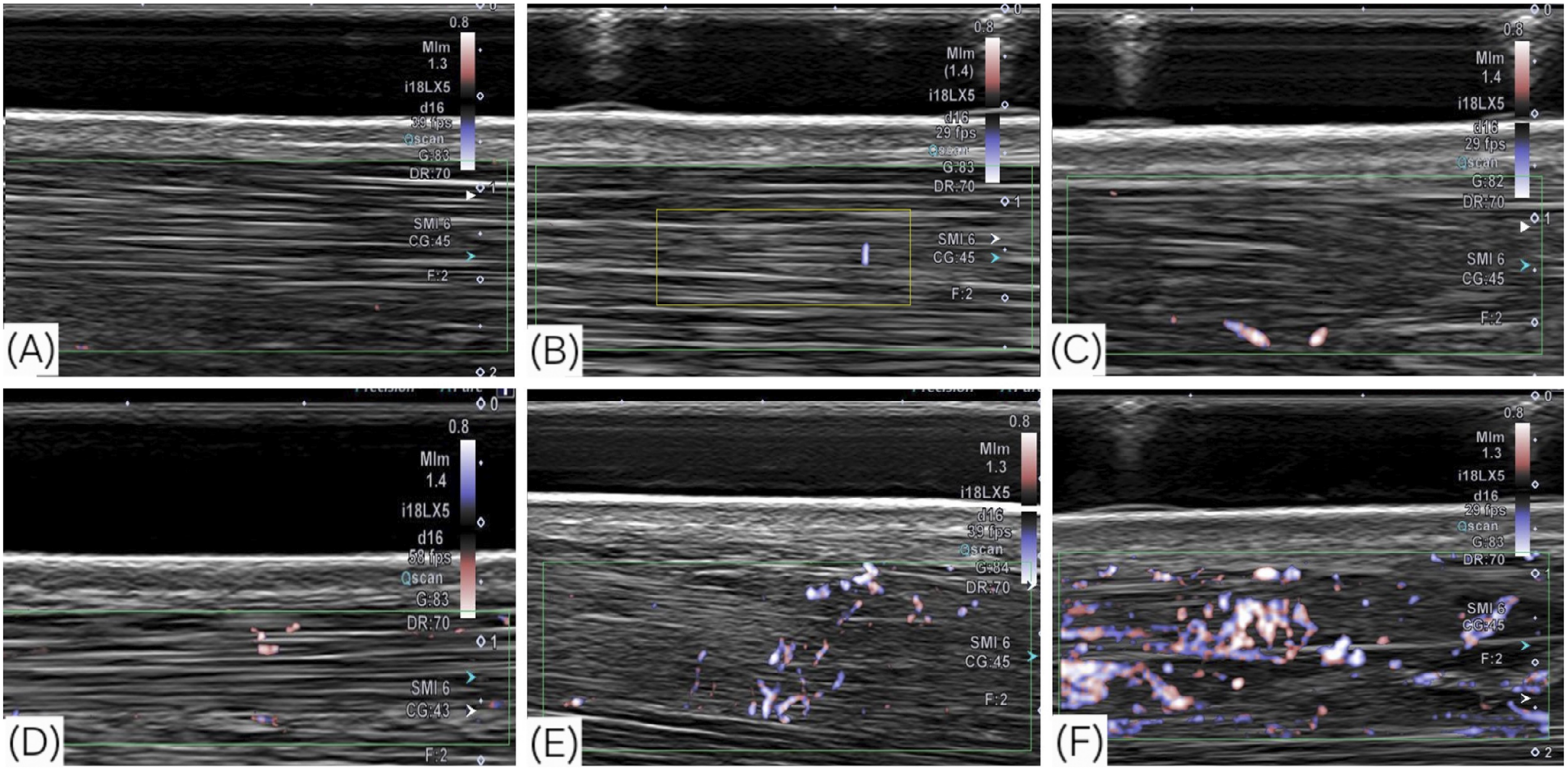
Representative weight‐bearing MVI (microvascular imaging) longitudinal images obtained with a stand‐off illustrating the adapted semiquantitative vascularity grading scale (Grades 0–5) used for evaluation of SDFT vascularization: (A) Grade 0; (B) Grade 1; (C) Grade 2; (D) Grade 3; (E) Grade 4; and (F) Grade 5.

**FIGURE 2:**
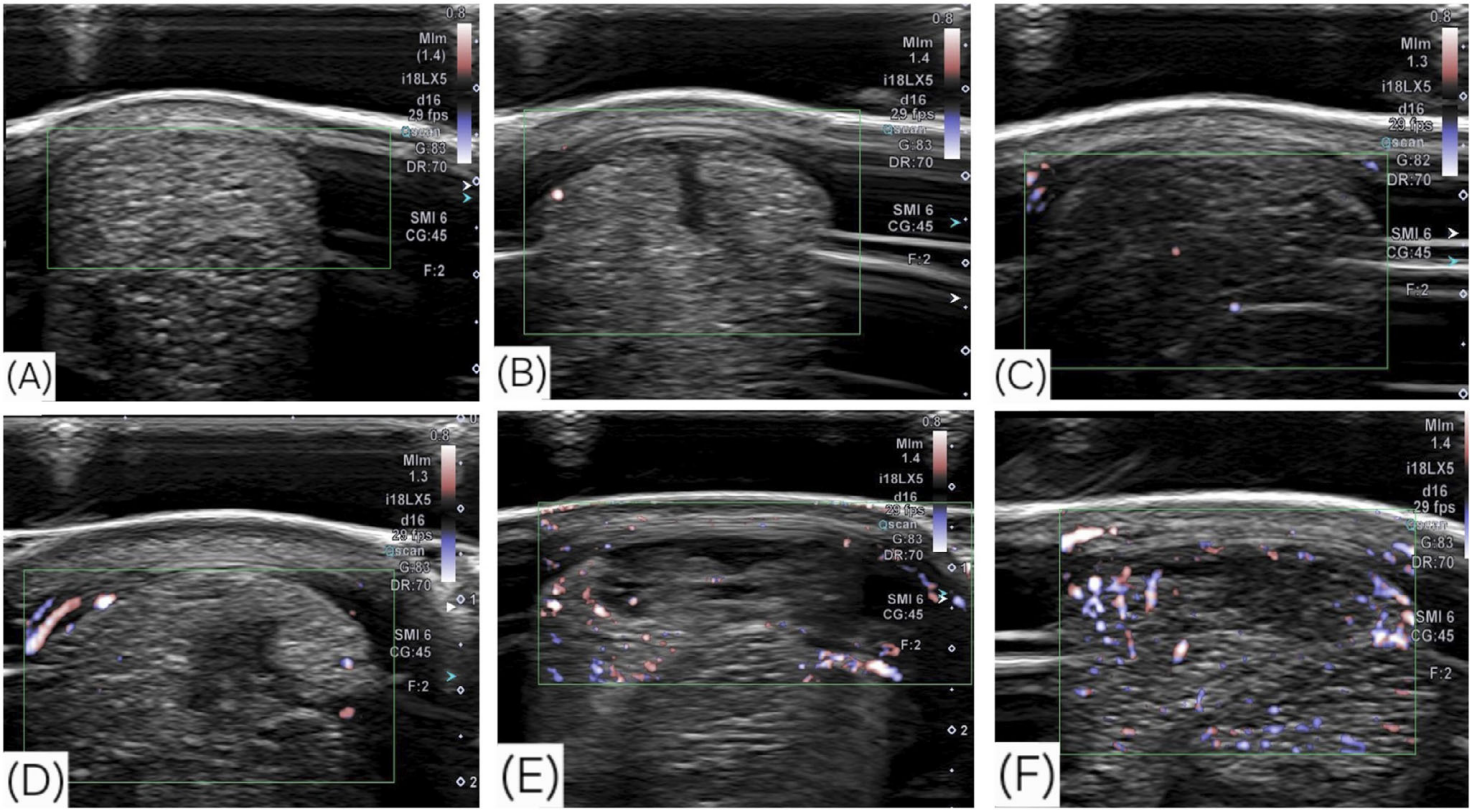
Representative weight‐bearing MVI (microvascular imaging) transverse images obtained with a stand‐off illustrating the adapted semiquantitative vascularity grading scale (Grades 0–5) used for evaluation of SDFT vascularization: (A) Grade 0; (B) Grade 1; (C) Grade 2; (D) Grade 3; (E) Grade 4; and (F) Grade 5.

Vascularity scores were obtained by blinded semiquantitative evaluation performed by two observers, one experienced (C.U., 20 years of equine ultrasound experience) and one inexperienced (M.G.C., 3 years of clinical experience), using a 0–5 scoring system (Table 1, Figures 1 and 2) adapted from Öhberg et al. [24] and Lacitignola et al. [12]. Quantitative analysis was performed on the previously exported still frames using ImageJ software (National Institutes of Health, USA) with a custom macro applied consistently across all images. A manual ROI was drawn over the visible tendon area on each image, and colored pixels within the ROI were identified using fixed RGB‐based thresholds applied uniformly across all images. Each processed image was manually reviewed to verify that identified pixels represented true vascular signal and that color artifacts were not misclassified. The number of colored pixels was then expressed as a ratio relative to the total tendon area, providing a standardized measure of vascularity. This approach provided an objective and reproducible measure of tendon vascularization, allowing for comparison over time [25].

**TABLE 1:** Semiquantitative vascularity grading system.

Grade	Adapted semiquantitative scale
0	No flow
1	One vessel or a single spot throughout the tendon
2	Two vessels throughout the tendon
3	Three vessels throughout the tendon
4	>Three vessels throughout the tendon
5	≥50% of tendon length or CSA with active vessel flow

### 2.3 Statistics

Descriptive analyses included computation of medians and ranges of continuous variables and tabulation of categorical variables. Tests of normal distribution (Shapiro–Wilk test) were performed to determine the extent of skewness of continuous data. Frequency counts and percentages were used for summarizing categorical variables.

Vascular signal scores not only exhibited an exponential distribution but also closely aligned with count data (Table 1), as increases in score corresponded to increases in the number (count) of observed vessels with flow. Scores were, therefore, modeled using mixed‐effects Poisson regression, with random effects used to account for repeated measurements within horses across weeks, imaging modalities, observers, and scanning planes. By contrast, vascular signal ratios were modeled using mixed‐effects gamma regression because they were continuous, positive, and right‐skewed outcomes, for which the gamma distribution is well suited. Because the gamma distribution is defined only for values greater than zero, ratio values of 0 were replaced with 0.0001 before analysis. Fixed effects for both models included imaging modality (MVI vs. PD), time as categorical variable (Weeks −1, 0, 2, 4, 8, 12), observer experience (expert vs. nonexpert), imaging plane (longitudinal vs. transverse), stand‐off use (SD vs. NSD), weight‐bearing condition (WB vs. NWB), limb (treated vs. control), and anatomical level (12, 16, and 20 cm DACB). Horse was included as a random effect to account for repeated measurements and unbalanced dataset. Analyses included 2164 observations for the semiquantitative and 1080 for the quantitative dataset.

Post hoc marginal (model‐adjusted) means and effects were calculated. The Holm–Šídák method was used to adjust the resulting *p* values for multiple comparisons. All marginal means and effects were reported with their respective 95% confidence intervals (95% CI, calculated based off standard error of the mean). All vascularity scores reported in the text represent model‐adjusted marginal means from the mixed‐effects Poisson regression model unless otherwise stated. All analyses were performed in Stata (StataCorp, College Station, TX) with two‐sided tests of hypotheses and statistical significance set at *p* < 0.05.

## 3 Results

### 3.1 Ultrasonographic Modality Performance

**FIGURE 3:**
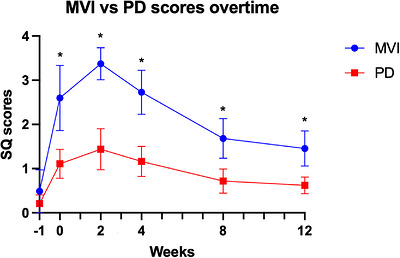
Model‐adjusted marginal mean semiquantitative vascularity scores (SQ scores) of the SDFT obtained using MVI (microvascular imaging) and PD (Power Doppler) at baseline (Week −1) and at Weeks 0, 2, 4, 8, and 12 following collagenase‐induced tendon injury. Asterisks indicate statistically significant pairwise differences between imaging techniques at the same time point. Error bars represent the 95% confidence intervals.

Semiquantitative vascularity scores were higher when using MVI versus PD at all time points (*p* < 0.001 for overall modality effect), except Week −1 (normal tendons). Post hoc pairwise comparisons confirmed statistically significant differences between MVI and PD at each individual post‐injury time point (Figure 3). PD demonstrated the same temporal pattern as MVI but with a lower vascularity score. The marginal mean PD scores (0.87, 95% CI: 0.64–1.10) across all weeks were approximately 55% lower than the corresponding MVI scores (2.04, 95% CI: 1.73–2.34; Figure 3). Compared with baseline (Week −1), vascularity scores at Weeks 8 and 12 for PD (*p* = 0.06 for Week 8 and *p* = 0.6 for Week 12) and at Week 12 for MVI (*p* = 0.44) were not significantly different from baseline. All other time points demonstrated higher vascularity scores for both PD and MVI (*p* < 0.05) when compared with baseline. The greatest increase was observed at Week 2 (3.37, 95% CI: 3.01–3.73 for MVI and 1.44, 95% CI: 0.97–1.90 for PD), when vascular activity was maximal (Figure 3). This temporal pattern was consistent across horses and imaging modalities and reflected concurrent changes observed on B‐mode imaging. Semiquantitative vascularity scores obtained with MVI and PD were strongly associated with echogenicity and fiber pattern scores across all interactions (*p* < 0.001). Detailed B‐mode findings have been reported previously [22]. Model‐adjusted analysis showed no effect of imaging level (16 and 20 cm from DACB, relative to the 12 cm reference; *p* = 0.16 and *p* = 0.6, respectively) or treatment (*p *= 0.7) on vascularity scores for either imaging modality.

**FIGURE 4:**
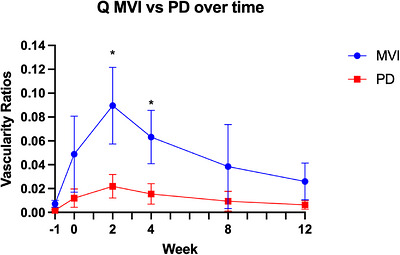
Model‐adjusted marginal mean vascularity ratios of the SDFT obtained using MVI (microvascular imaging) and PD (Power Doppler) at baseline (Week −1) and at Weeks 0, 2, 4, 8, and 12 following collagenase‐induced tendon injury. Asterisks indicate statistically significant pairwise differences between imaging techniques at the same time point. Error bars represent the 95% confidence intervals.

Quantitative vascularity ratios were higher with MVI than PD at all time points (*p* < 0.001 for overall modality effect), except at Week −1 (normal tendons). Post hoc pairwise comparisons confirmed significant differences between MVI and PD at the peak post‐injury time points of Weeks 2 and 4 (*p* < 0.001 and *p* = 0.002, respectively; Figure 4). PD demonstrated the same temporal pattern as MVI but with lower vascularity ratios. The marginal mean PD ratios (0.011, 95% CI: 0.007–0.015) across all weeks were approximately 75% lower than the corresponding MVI ratios (0.046, 95% CI: 0.030–0.060; Figure 4). Compared with baseline (Week −1), MVI ratios were not significantly different at Weeks 0 and 12 (*p* = 0.321 and *p* = 0.754, respectively), whereas PD ratios were not significantly different at Weeks 8 and 12 (*p* = 0.055 and *p* = 0.581, respectively). All other time points demonstrated higher vascularity ratios for both modalities (*p* < 0.05).

### 3.2 Scanning Technique

Semiquantitative vascularity scores were significantly lower in the transverse plane (0.92, 95% CI: 0.69–1.15) compared with the longitudinal plane (1.97, 95% CI: 1.72–2.22, *p* < 0.001), representing a 53% lower vascularity score in the transverse orientation for MVI and PD imaging modalities. Quantitative vascularity ratios were also significantly lower in the transverse plane (0.018; 95% CI: 0.005–0.031) compared with longitudinal plane (0.039; 95% CI: 0.035–0.042), representing a 54% reduction in vascularity ratio (*p *< 0.001).

**FIGURE 5:**
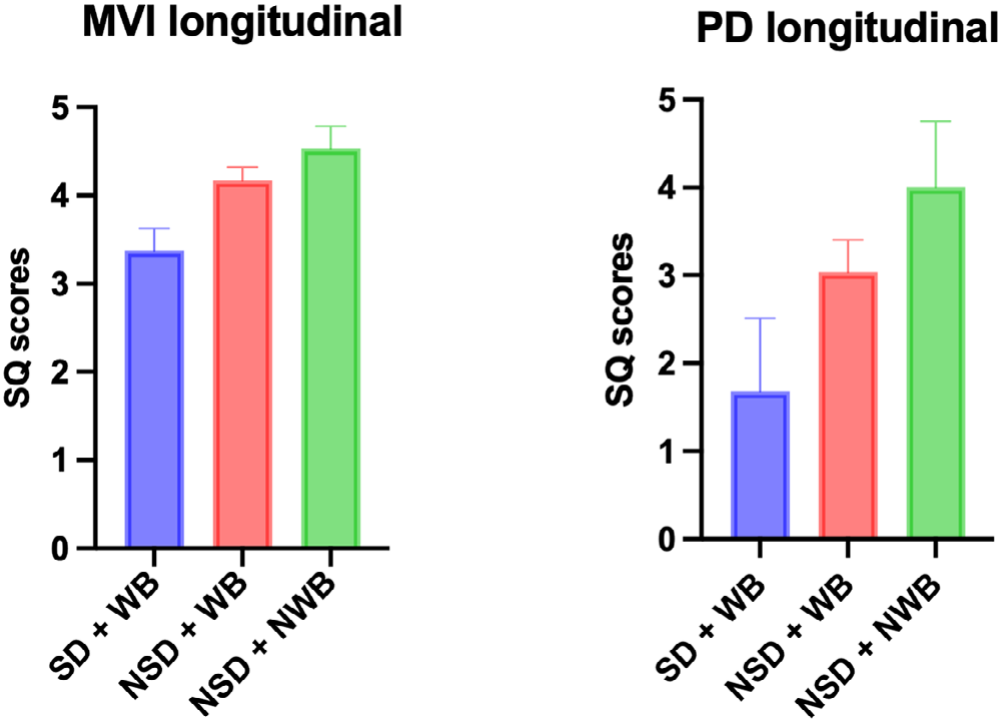
Effect of scanning technique on vascular signal detection in the longitudinal plane using MVI (microvascular imaging) and PD (Power Doppler). Columns represent median semiquantitative vascularity scores (SQ scores; Grades 0–5) for images acquired under different scanning conditions: weight‐bearing (WB) versus non‐weight‐bearing (NWB) stance and with (SD) or without (NSD) use of a stand‐off pad. The SD + NWB combination was not performed in this study. Error bars indicate the interquartile range.

The vascularity scores for horses in the WB and NWB stance, with and without use of an SD, are shown in Figure 5. Although differences were not statistically significant, WB scans demonstrated marginal predicted vascularity scores that were slightly lower than those obtained in the NWB stance (1.49, 95% CI: 1.30–1.69 vs. 1.72, 95% CI: 1.36–2.08, respectively*, p *= 0.176). Similarly, no difference was observed between quantitative vascularity ratios in the WB and NWB stance (0.03; 95% CI: 0.02–0.04 vs. 0.04; 95% CI: 0.01–0.06, respectively, *p = *0.775). There was no statistical difference between use of an SD and NSD for both vascularity scores (1.49, 95% CI: 1.29–1.70 vs. 1.60, 95% CI: 1.20–1.98, respectively, *p* = 0.648, Figure 5) and quantitative vascularity ratios (0.024; 95% CI: 0.02–0.03 vs. 0.07; 95% CI: 0.01–0.12, respectively, *p* = 0.108).

### 3.3 Observer Experience

Observer experience significantly influenced the degree of vascular signal detected in the semiquantitative score grading (*p* < 0.001). The inexperienced observer (1.38, 95% CI: 1.15–1.60) consistently assigned lower vascularity scores than the experienced observer (1.64, 95% CI: 1.47–1.82, *p* < 0.001, respectively), with predicted marginal mean scores approximately 18% lower. Despite this difference in magnitude, both observer groups demonstrated the same overall pattern of semiquantitative vascular scores change across weeks and modalities. Interobserver variability related to operator experience was not assessed for quantitative analyses, as all images were analyzed once using a standardized ImageJ macro.

## 4 Discussion

This study demonstrates temporal changes in tendon vascularity after lesion induction in horses with collagenase‐induced SDFT tendonitis, with MVI detecting more vascularity than PD using both semiquantitative and quantitative assessments. Both imaging modalities (MVI and PD) demonstrated a consistent temporal pattern following lesion induction, characterized by an early increase in vascular signal (Week 0), a peak at 2 weeks, and a gradual reduction thereafter. PD returned closer to baseline earlier than MVI, likely due to its lower peak signal intensity. This pattern is consistent with angiogenesis and fibroplasia during the early proliferative phase of tendon healing, followed by tissue remodeling and scar maturation [12, 26].

**FIGURE 6:**
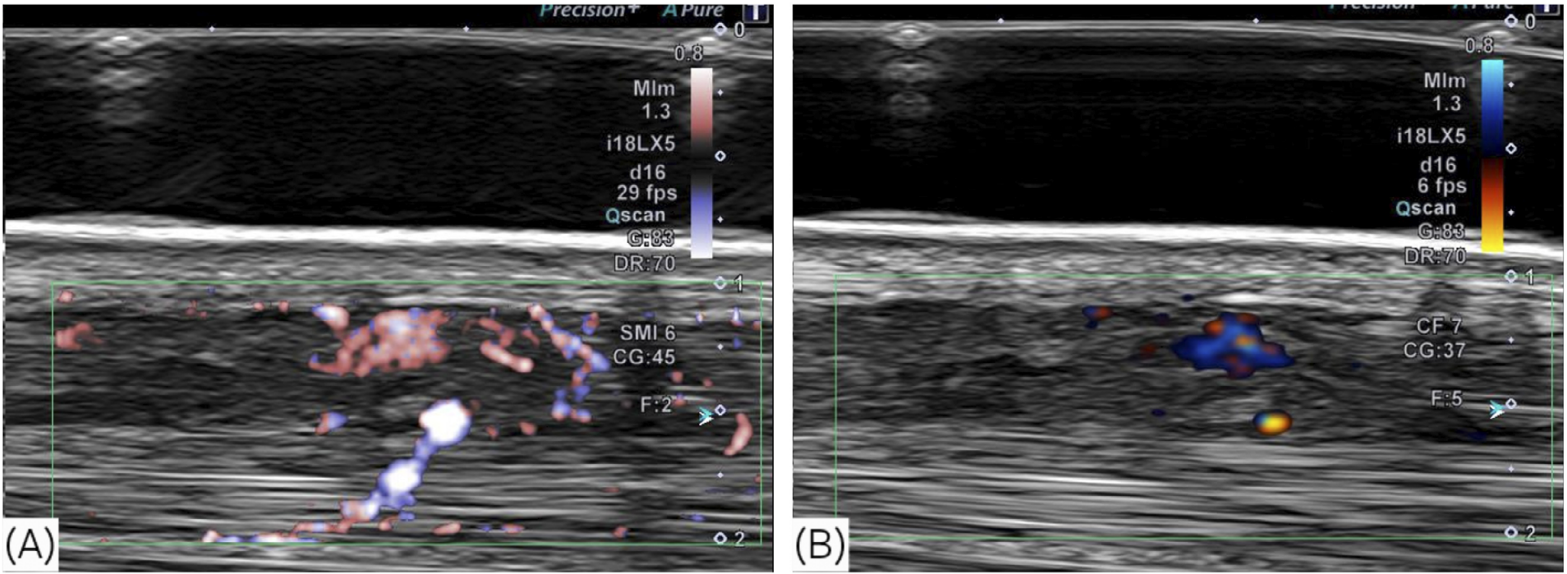
Corresponding representative longitudinal MVI (microvascular imaging) and PD (Power Doppler) images of the SDFT at 16 cm DACB in the right forelimb of one horse. Images were obtained at Week 2 in the weight‐bearing stance with a stand‐off pad: (A) MVI longitudinal image graded with 5 score; (B) PD longitudinal image graded with 3 score.

Across all time points except baseline, MVI detected more vascularity than PD, suggesting it is a more sensitive technique for assessing microvascular flow during tendon injury and rehabilitation (Figure 6). This is particularly pertinent during Weeks 8–12, when PD vascularity scores were not different from baseline and PD signal had returned to close to pre‐injury levels with a mean score of <1, suggesting that by this stage of healing, vascularity was no longer detected with PD at this time point (0.71, 95% CI: 0.44–1). In contrast, MVI scores at Week 8 remained significantly elevated above baseline (1.68, 95% CI: 1.23–2.13) (Figure 4). At Week 12, although there was no significant difference to baseline, the marginal mean score was >1, suggesting the presence of residual microvascular activity during later healing stages. These findings align with reports in human musculoskeletal imaging, where MVI has improved visualization of microvascularity in inflammatory and degenerative soft‐tissue conditions [17, 18, 21, 24, 27] and contributed to enhanced diagnostic and monitoring of rheumatic, tendinopathies, adhesive capsulitis, and carpal tunnel syndrome [20, 26]. In horses, these findings suggest that MVI has potential for detecting early tendon pathology, monitoring lesion progression, and responding to treatment. Moreover, human studies have demonstrated associations between MVI‐detected neovascularization and pain severity, supporting its potential role in differentiating chronic active versus chronic degenerative tendinopathies and desmopathies [20]. Whether similar associations exist in horses remains unknown and warrants investigation in naturally occurring equine tendinopathies.

Quantitative vascularity ratios mirrored the semiquantitative findings, further supporting the superiority of MVI over PD in detecting intratendinous flow. MVI vascularity ratios were higher than PD ratios at all post‐injury time points, reaching statistical significance at Weeks 2 and 4. This concordance between scoring methods strengthens confidence in the biological signal. Whereas the semiquantitative score relies on subjective observer grading, the pixel‐based ratio is derived from standardized image analysis and is therefore less susceptible to observer‐related bias. Both modalities exhibited a consistent temporal pattern, with vascularity peaking at Week 2 and declining progressively thereafter. Notably, MVI detected sustained significant elevations through Week 8, whereas PD did not identify significant changes beyond Week 4, suggesting lower sensitivity of PD during later stages of repair when flow velocities and microvascular volume are reduced. The parallel longitudinal trends support the interpretation that observed vascularity changes reflect true physiological processes in tendon healing rather than measurement artifacts.

Structural changes on B‐mode were reported on the previous publication [22], where echogenicity increased and fiber pattern improved over time, reflecting the transition from early edema and collagen disruption to later reorganization [8, 9, 12]. These B‐mode parameters correlated with both MVI and PD vascularity scores, although the association was stronger for MVI. This relationship between vascularity and structural abnormalities has been described in equine and human tendinopathy and supports the use of MVI as a complementary technique rather than a standalone diagnostic tool [8, 9, 13, 14].

The imaging plane had a significant effect on vascular detection. Transverse images yielded lower semiquantitative scores and quantitative ratios compared with longitudinal images, likely due to two complementary mechanisms. Primarily, longitudinal imaging optimizes anatomical orientation by allowing vessels to be visualized along their entire length as elongated linear structures. These are easier to detect and quantify than the small focal signals observed in transverse images, where differentiation from background noise or imaging artifacts is also more challenging. Additionally, tendon anisotropy may alter backscatter with changes in insonation angle, thereby reducing detection of small moving reflectors in transverse images, although this effect is less likely with an experienced sonographer [28]. This finding is consistent with the author's qualitative impression in clinical cases and has important practical implications, particularly if vascularity is being quantified, and suggests that longitudinal views may provide more consistent evaluation of tendon vascularity in the equine metacarpus. Concurrent use of the transverse image, however, is often beneficial for easier localization of neovascularization relative to both pathology and normal anatomic structures.

WB and NWB, as well as the use of an SD, had measurable but generally nonsignificant effects on semiquantitative or quantitative vascularity measurements. Although differences did not reach statistical significance, scans performed with NSD pad and in an NWB position tended to yield higher vascularity scores, representing clinically relevant differences (Figure 6). The lack of statistical significance is likely attributable to low numbers and limited statistical power, particularly as the number of NWB acquisitions was reduced by poor compliance with leg lifting. The observed differences may therefore be detectable in a future prospective and more adequately powered study. Posture when WB and the duration of NWB positioning may also influence Doppler signal intensity and hence should be considered. However, these effects have not been previously described, nor was the present study designed to investigate them. The reduction in vascular signal while standing is likely due to microvascular occlusion caused by increased tension during WB and the increased mechanical forces applied when using an SD as previously hypothesized [14]. Stand‐off pads may also reduce vascular signal by altering the acoustic beam path, near‐field conditions, and signal attenuation [29]. Although removal of the stand‐off pad is straightforward, maintaining an NWB position can be physically demanding and challenging and is occasionally not feasible in poorly compliant horses (as seen in this study). Therefore, on the basis of these findings and clinical experience, the authors routinely acquire MVI images in a longitudinal WB stance with NSD, progressing to an NWB stance if there is minimal flow in a suspected B‐mode lesion on WB images. However, further work is needed to determine optimal acquisition protocols.

Observer experience significantly influenced semiquantitative vascular scoring. The inexperienced observer consistently assigned lower absolute scores but followed the same temporal trends as the experienced observer. This pattern is consistent with established observer dependence in musculoskeletal ultrasound, particularly in Doppler techniques where small variations in gain, probe pressure, or incidence angle can affect apparent vascular signal. The semiquantitative grading scale shifts from discrete vessel counts (Grades 1–4) to an area‐based criterion at Grade 5 to accommodate diffuse MVI signals. This transition may introduce scoring inconsistencies, with observers reporting the greatest difficulty distinguishing Grades 4 and 5, likely contributing to the interobserver variability observed. In future studies, the addition of a category for 25%–50% of tendon length or cross‐sectional area (CSA) with active vessel flow would be recommended. Despite this, both observer groups reliably identified relative changes over time, suggesting that temporal trends may be robust to differences in experience, underscoring the objectivity and reproducibility of ratio‐based vascularity assessments.

Multiple limitations should be acknowledged. The small sample size constrained some statistical models and resulted in occasional non‐convergence. Vascular signals detected by MVI and PD were not confirmed histologically, so it cannot be excluded that some of MVI's additional signal reflects artifact rather than true microvessels. From a methodological standpoint, as an intralesional treatment comparison (APS vs. saline) was not statistically significant, data from both forelimbs were pooled to maximize statistical power. Additionally, the collagenase‐induced model produces a chemically mediated injury that may not fully reproduce the pathophysiology of naturally occurring tendinopathy. All examinations were performed by a single sonographer, ensuring consistent acquisition technique but precluding assessment of inter‐operator reproducibility. Still‐frame selection was performed by one individual prior to blinded scoring, which may have introduced bias. Future studies should consider automated or independent frame extraction. The pixel‐based vascularity ratio used identical RGB thresholds across MVI and PD images despite their different color maps and should therefore be regarded as a complementary metric to the semiquantitative scores rather than a standalone measure. Finally, despite statistical adjustments, factors, such as incomplete scanning technique, limb positioning, and physiologic variability, may have influenced vascular measurements and comparisons. Currently, limitations of MVI in general include the lack of standardized acquisition protocols, variability among ultrasound systems, and limited availability in some clinical settings, which may restrict broader clinical adoption and highlight the need for further investigation.

In conclusion, this study provides the first characterization of intratendinous vascularity using MVI in an equine model of SDFT injury, demonstrating that MVI represents a valuable adjunct to conventional ultrasonography for the diagnosis and monitoring of tendinopathies. Although further work is required, longitudinal images performed without a stand‐off showed promise as an optimal acquisition technique. Future studies involving horses with naturally occurring tendinopathies will be essential to better characterize the MVI patterns during lesions development and healing.

## Author Contributions

**Joie L. Christensen**: writing – review and editing, software, data curation. **Darko Stefanovski**: writing – review and editing, formal analysis, software, data curation. **Miriam R. Garcia‐Collao**: writing – original draft, investigation, methodology, project administration, software, conceptualization, formal analysis, data curation, visualization, writing – review and editing. **Kyla Ortved**: writing – review and editing, validation, supervision, investigation, conceptualization, methodology. **Claire Underwood**: project administration, conceptualization, investigation, validation, supervision, writing – review and editing, visualization, methodology.

## Conflicts of Interest

The authors declare no conflicts of interest.

## Data Availability

The raw data supporting the conclusions of this article will be made available by the authors, without undue reservation.
